# External context in individual placement and support implementation: a scoping review with abductive thematic analysis

**DOI:** 10.1186/s13012-023-01316-w

**Published:** 2023-11-09

**Authors:** Jaakko Harkko, Noora Sipilä, Hilla Nordquist, Tea Lallukka, Kaija Appelqvist-Schmidlechner, Michael Donnelly, Anne Kouvonen

**Affiliations:** 1 Faculty of Social Sciences, University of Helsinki, Helsinki, Finland; 2 The Equality Unit, Finnish Institute for Health and Welfare, Helsinki, Finland; 3 South-Eastern Finland University of Applied Sciences, Kotka, Finland; 4 Department of Public Health, University of Helsinki, Helsinki, Finland; 5 Centre for Public Health, Queen’s University Belfast, Belfast, Northern Ireland

**Keywords:** Implementation, Scale-up, Evidence-Based Health Care, Psychiatric disorders, Supported employment, Delivery of Health Care

## Abstract

**Background:**

Context including the external context may considerably affect the adoption, implementation, sustainment, and scale-up of evidence-based practices. We investigated external contextual features by conducting a scoping review of empirical research regarding the implementation of an evidence-based psychiatric or mental health vocational rehabilitation service called Individual Placement and Support (IPS).

**Methods:**

The protocol for the scoping review was registered with the Open Science Framework. We used the methodology by Joanna Briggs Institute for conducting the scoping review and reported it according to the PRISMA-ScR checklist. We searched 12 databases for research regarding ‘Individual Placement and Support’ or ‘Evidence-Based Supported Employment’. We retained peer-reviewed empirical studies investigating external contextual factors and their impact on IPS implementation outcomes. We extracted data from the eligible articles and conducted descriptive and thematic analyses.

**Results:**

Fifty-nine original research papers met our eligibility requirements and were retained after reviewing 1124 titles and abstracts and 119 full texts. The analysis generated two main themes: (1) external contextual determinants of service delivery and (2) external systems influencing the evidence-to-practice process. The first main theme encompassed policies and laws, financing, and administratively instituted support resources, and organizational arrangements associated with external stakeholders that may facilitate or hinder the local implementation. The second main theme comprised strategies and actions used by different stakeholders to facilitate implementation locally or scale-up efforts at a system level.

**Discussion:**

Our scoping review illustrates the important role that external contextual factors play and how they may facilitate or hinder the implementation and scale-up of the IPS model across mental health services in different countries. Consideration of these factors by decision-makers in mental health and welfare services, planners, providers, and practitioners is likely to facilitate the development of effective strategies for bridging the evidence-practice gap in implementing the EBPs. Finally, the scoping review identified gaps in knowledge and offered suggestions for future research.

**Trial registration:**

Open Science Framework

**Supplementary Information:**

The online version contains supplementary material available at 10.1186/s13012-023-01316-w.

Contributions to the literature

- The scoping review of 59 studies provides a summary of results from empirical implementation studies covering the implementation, sustainment, and scale-up of the Individual Placement and Support (IPS) model, evidence-based practice in mental health services to obtain employment for persons with mental disorders.
- The study identifies external contextual factors occurring consistently across the reviewed literature.
- The study highlights the strategies and actions that different key stakeholders (namely researchers, political and administrative decision-makers, support organizations, and agency leaders) undertake to facilitate or hinder the local implementation and scale-up efforts at the system level.

## Background

The institutions and structures outside a service organization can significantly impact the implementation of evidence-based practices (EBPs). These contextual factors can affect both the implementation strategies [1] and outcomes [2], including the sustained use of EBPs [3]. Factors such as scientific support, funding, legislation, social policy, supportive educational and training structures, variables related to communities, the service environment, leadership, and networks have been identified as crucial for translating evidence into practice [4–7]. These external context factors have also been recognized as important targets for systematic study [4, 7], and the lack of emphasis on the system and policy levels of implementation has been considered to contribute to suboptimal results in translating evidence to practice [8]. However, the concept ‘external context’, which largely overlaps in meaning with other concepts such as ‘outer setting’ or ‘external environment’, is defined ambiguously and inconsistently across various studies [9, 10]. It also is less frequently the focus of empirical observation in implementation and dissemination research [2, 11, 12]. For these reasons, there is a need to increase the efforts to organize and systematize the findings from existing research literature in a structured way.

In this scoping review, we examined the processes, mechanisms, and social systems traditionally considered as ‘external context’ in relation to the implementation of the Individual Placement and Support (IPS) model. IPS is an EBP in mental health care that integrates vocational rehabilitation and mental health treatment through a multidisciplinary team approach [13]. Meta-analyses have shown that the IPS model effectively supports people with mental disorders to paid employment [14, 15]. The model’s feasibility for different patient groups and the predictive validity of the fidelity model has also been demonstrated [16, 17]. The model was developed in the USA in the 1990s and is now used in many countries, including the USA, Canada, Australia, and several European countries. In addition to building up the evidence base, the model’s creators’ have engaged in several ways to increase the model’s penetration and reach. These efforts include the IPS Learning Collaborative, a two-tier dissemination model for administration representatives and regional support organizations [18, 19]. The model’s originators also have produced standardized guidelines and training materials, published standards for monitoring the implementation quality, participated in training state trainers, and provided summaries of the evaluation and monitoring reports [18, 19]. Despite these efforts, the IPS model has achieved relatively low penetration in service systems across countries [20–22].

The motivation to study the external context, specifically with respect to the IPS model, is driven by several converging reasons. First, there is a noticeable overlap in time between the maturing evidence base and reports of challenges in innovation dissemination. Second, since the late 2000s, there has been a growing body of individual studies that identify external contextual factors as barriers affecting the implementation and penetration of IPS. This review attempts to identify potentially consistent trends across existing research findings. Third, the IPS model promotes the ‘recovery approach’. This approach values community inclusion as a pivotal aim of the care process [23, 24]. The recovery approach signifies a society-driven shift in the care paradigm, a shift that is inherently connected to the evolution of the service system which is part of the ‘external context’. Finally, studying a relatively consistent and homogenous intervention may reduce the variability that may occur when summarizing and comparing results from interventions with different foundational principles or methodologies, i.e., differences between interventions may act as confounding variables.

Previously, only one study has attempted to systematically review empirical research on external context constructs that affect the implementation of complex evidence-based health interventions [2]. We searched the Cochrane Database of Systematic Reviews and Joanna Briggs Institute (JBI) Evidence Synthesis but identified no current or underway systematic or scoping reviews on our topic. We chose a scoping review and not systematic review methodology as the scope of our review is the context rather than the properties of the intervention, and as the concept ‘external context’ is not unambiguously defined or measured in literature, and the purpose of this study is to discuss the implementation science concepts [23–25].

### Objectives

We conducted a scoping review to systematically map the empirical research covering the external contextual factors in implementing and scaling up the IPS model and identify existing gaps in knowledge. Our research question was ‘How does external context affect the adoption, implementation, sustainment, and scale-up of the Individual Placement and Support (IPS) Model?’.

## Methods

### Protocol and registration

We followed the JBI methodology [23, 26] to produce the protocol. We prospectively registered the protocol with the Open Science Framework [27].

### Eligibility criteria

We followed the ‘Population/Concept/Context’ framework (PCC) recommended by JBI [23] for scoping reviews. We defined our study population to encompass all pertinent stakeholders, including practitioners, researchers, policymakers, state bureaucrats, and leaders of mental health agencies. The concept examined by this scoping review was ‘external context’ which we define as any condition or circumstance external to the agency where the IPS model is executed, as outlined in the model guidelines [28]. We define context along this distinction as ‘local service context’ and ‘external context’.We accepted studies with no country restrictions. We included only peer-reviewed journal articles covering IPS services targeted at persons with any mental disorder written in English. Quantitative, qualitative, and mixed-method studies were included. We excluded studies not meeting the eligibility criteria, e.g., non-English studies, gray literature, theses and conference abstracts, and theoretical studies.

### Information sources

We searched the following bibliographic databases: PROSPERO, the Cochrane Database of Systematic Reviews and the JBI Database of Systematic Reviews and Implementation Reports, and APA PsycInfo, Pubmed, Science Direct, ProQuest Social Science Premium Collection (Sociology Collection, Social Science Database, Politics Collection), and Ebsco Psychology/Sociology Databases (CINAHL, SocINDEX, Academic Search Complete). We used assistance from university librarians in choosing the databases.

### Search

We conducted database searches using the search terms ["Individual Placement and Support"] and ["Evidence-based supported employment"] individually in all databases. We limited our search to study titles and abstracts and, when possible, selected the option to include only peer-reviewed articles. We conducted the searches first in April 2022, and the searches were updated in January 2023. Our search was restricted to articles published up to December 2022, with no restrictions for the earliest publication dates. We further employed the snowballing technique by reviewing the reference lists of all the studies included after the screening. The number of screened studies was 1 124 in total. The review of additional articles from the reference list search did not lead to any changes in the established theme structure, and no further searches were conducted.

### Selection of sources of evidence

The titles and abstracts were uploaded into Covidence systematic review software (Veritas Health Innovation, Melbourne, Australia). The screening was undertaken independently by two reviewers. The first author and one of the second reviewers (NS, HN, TL, KA-S, AK) assessed the titles and abstracts against the eligibility criteria. Full texts were reviewed when screening produced indecisive results. We solved potential disagreements about study selection with a consensus method with three reviewers. We applied a similar procedure for full-text screening.

### Data charting process

The first author created the list of data-charting items and initial code structure to determine the units of analysis to be extracted. The list was updated during the analysis iteratively to produce the best obtainable data description. The process was conducted in collaboration with the co-authors.

### Data items

The first author undertook data extraction. The extracted data on article characteristics included year of publication, geographical area, aims, population, methods, and main results. For further thematic analysis, we extracted all text in the results sections of the articles that referred to ‘external context’ and could be associated with those ‘implementation outcomes’ referring to the extent to which the innovation has been implemented or is being delivered [29, 30]. They include adoption, implementation, and sustainment, which refer to local ‘actual implementation outcomes’ [30], as well as penetration and reach, which pertains to corresponding ‘actual’ system-level implementation outcomes. Only sections covering the IPS model were extracted if the articles included observations from multiple EBPs. Following the Preferred Reporting Items for Systematic Reviews and Meta-Analyses Extension for Scoping Reviews (PRISMA-ScR) guidelines [25], we did not conduct quality assessments for the articles.

### Synthesis of results

The first author charted, extracted, and classified the data using Atlas.ti (version 9.1.7) software. The extracted data was subjected to abductive thematic analysis. Abductive thematic analysis is a research approach that combines inductive and deductive reasoning to iteratively explore and interpret data, aiming to generate the most plausible explanations for observed phenomena by aligning empirical findings with existing theoretical frameworks or creating new ones [31, 32]. We used an existing and widely used theoretical framework (Consolidated Framework for Implementation Research, CFIR) as the starting point of the data coding. However, we used an inductive approach for categorization and thematization when the empirical data was considered relevant, but the framework did not provide a straightforward way to classify the items or patterns observed in the data. This approach led us to use a two-stage approach to presenting the results. First, we report the results describing the ‘outer setting domain’ [4, 33] determinants of the ‘actual implementation outcomes’ [30], i.e., the results congruent with the CFIR framework. This section is labeled ‘External contextual determinants’ and describes the data in which each text excerpt representing a determinant and implementation outcome association was coded as either ‘Facilitators’ or ‘Barriers’. In this section, we also present those ‘inner setting’ items that may be evaluated as subject to external societal and professional influences or were reported as targets of interventions by the external stakeholder. Second, we describe results associated with the strategies and actions of the different external stakeholders under the label ‘Systems of evidence-to-practice’. These ‘resource systems’ [34] encompass organizations and individuals that shape the external determinants of local implementation or aim at system-level implementation outcomes. Informed by the socio-ecological [7, 35, 36] and complex adaptive [37] systems approaches these ‘resource systems’ were perceived as multi-layered, self-organizing, interacting with each other, with outcomes that are contingent and intrinsically uncertain. We organized the results by stakeholder groups and presented the associations of these items with other external contextual determinants.

We convey the findings narratively, highlighting the key aspects and findings of each category. As the thematic categories utilized in our study were mutually non-exclusive and the same data excerpts could be coded with several codes, we avoid double reporting of the items when possible. We present results according to PRISMA-ScR [25], and the completed checklist is in Additional file 1. Descriptive tables were compiled with Stata Statistical Software (Version 17). We used Grammarly (www.grammarly.com) and OpenAI’s Chat-GPT 3 (https://chat.openai.com/chat, version 13) for proofreading purposes.

## Results

### Selection of sources of evidence

**Fig. 1:**
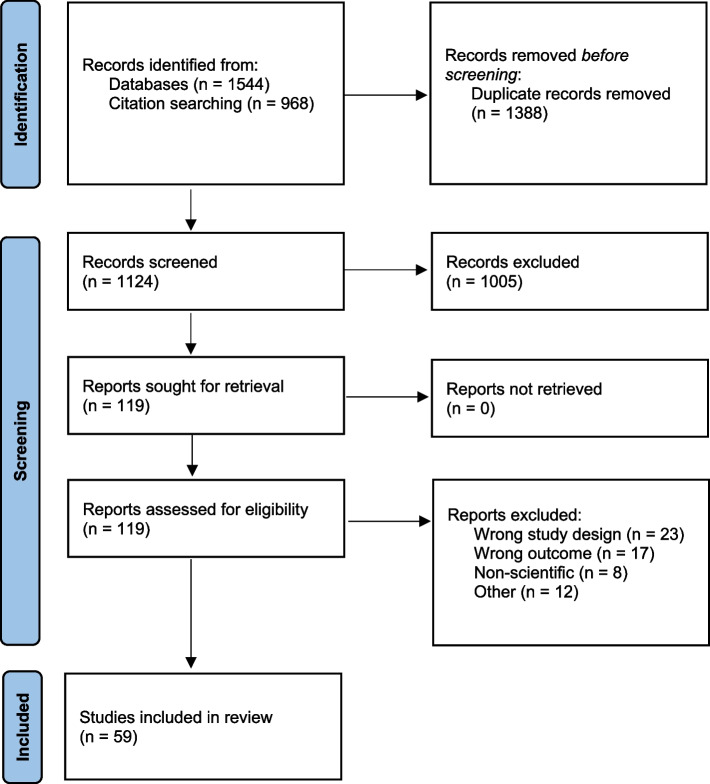
PRISMA flow diagram

The search and screening results at each stage are shown as a PRISMA flow chart in Fig. 1. We screened unique 1124 titles and abstracts and 113 full-text documents. Of those, 59 original research papers met the eligibility criteria. The full list of included studies can be found in Additional file 2, and the record of excluded full-text studies can be found in Additional file 3.

### Characteristics of sources of evidence

Eight out of 59 included studies used quantitative methodologies, 33 qualitative, and 18 mixed methods. Twenty-four were conducted in the USA, 7 in the Netherlands, 6 in Sweden, and 4 in Canada, Australia, and England. The remaining 10 studies were conducted in other countries. The year of publication ranged from 1998 to 2022 (median = 2017). The study aims reflected high variability in the scope of investigations, ranging from those interested in implementation processes and evaluation, through stakeholder perspectives and experiences, to comparative and regional analysis. Descriptive data about the included studies are presented in Table 1.

**Table 1:** Descriptive characteristics of included studies

Article	Country	Aims	Population	Methods	Main results
i1^*^. Bakkeli ... (2022)	Norway	To explore how evidence-based standards are ‘made to work’ by frontline workers and managers in everyday service provision	Agency leaders/managers, staff	Qualitative	IPS standards may be implemented in joint employment services in ways that may either promote more radical change or revive traditional practices
i2. Becker ... (1998)	USA	To identify areas that are critical for successful implementation	Support organization, agency leaders/managers	Qualitative	Successful implementation is facilitated by leadership, organizational structure, training, finances, and time frames
i3. Becker ... (2007)	USA	To identify differences in access to supported employment services and rates of competitive employment	Agency leaders/managers	Quantitative	Financing was reported as the most critical predictor of the model’s reach
i4. Becker ... (2007)	USA	To explore factors associated with access to high-quality SE services	Agency leaders/managers	Qualitative	Diverse state-level strategies may facilitate the implementation of IPS services
i5. Bejerholm ... (2011)	Sweden	To illustrate the IPS approach in the Swedish welfare system	Support organization, agency leaders/managers, Staff	Qualitative	The results showed that the IPS principles were challenged by the welfare system, with differences in work capacity and type of welfare benefit impacting IPS delivery, leading to frustration among professionals
i6. Bergmark ... (2018)	Sweden	To describe and analyze barriers and facilitators for implementation	Political/administrative decision-makers, support organization, other external stakeholders, agency leaders/managers, staff	Mixed	Strategic networking, as well as the need for planning and preparations carried out before the start of an EBP program facilitate the adoption
i7. Bergmark ... (2019)	Sweden	To analyze the implementation and sustainability of evidence-based community mental health services	Other external stakeholders, agency leaders/managers, staff	Mixed	Rigorous preparation, including planning for collaboration, financing, and assessments of program fidelity, is particularly beneficial for implementing agencies before the start of a program, especially with regard to organizational level circumstances
i8. Bond ... (2008)	USA	To describe the implementation of supported employment in the National Evidence-Based Practices Project	Agency leaders/managers, staff	Mixed	High fidelity of implementation is attained by affirmative leadership decisions
i9. Bond ... (2017)	USA	To examine the activities of leaders in 13 states that have successfully implemented, sustained, and expanded evidence-based supported employment	Political /administrative decision-makers	Quantitative	Leaders in 13 states participating in a learning community have adopted and maintained multiple strategies to sustain and expand evidence-based supported employment at a high level of fidelity with good employment outcomes
i10. Bond ... (2021)	USA	To compare two states that have implemented with success (adopting states) and two that have faced challenges (non-adopting states)	Political/administrative decision-makers	Qualitative	Funding at the state level and collaboration between the state agencies determine implementation success
i11. Bonfils ... (2022)	Denmark	To examine the implementation of IPS through a case study of four IPS units	Agency leaders/managers	Mixed	The integration of IPS with mental health services was also found to be challenging as mental health services regarded IPS as a parallel service rather than a mutual responsibility
i12. Boyce ... (2008)	England	To assess the extent to which the Individual Placement and Support (IPS) approach is currently adopted in England	Agency leaders/managers, staff	Mixed	Constraints influencing providers’ ability to provide an IPS service were related to funding, values, and organizational policy
i13. Campbell ... (2007)	USA	To compare the fidelity of implementation of supported employment in different types of provider organizations	Document analysis/none stated	Quantitative	Community mental health programs rated significantly higher on fidelity than programs housed in psychosocial rehabilitation or comprehensive rehabilitation centers
i14. Carlsson ... (2022)	Sweden	To analyze implementation and de-implementation factors among Swedish municipalities that provide support to vulnerable clients through the Housing First (HF) or Individual Placement and Support (IPS)	Agency leaders/managers, staff	Mixed	Implementation barriers can be found at both the system and organizational levels, impacting front-line workers
i15. Cohen ... (2020)	USA	To examine the implementation and process evaluation of two types of Individual Placement and Support (IPS)	Document analysis/none stated	Qualitative	Barriers commonly encountered across provider sites included lack of leadership support, issues with agency structures and funding mechanisms, and difficulties in coordinating between child and adult systems
i16. Corbiere ... (2010)	Canada	To assess the implementation of SE services in three Canadian provinces	Agency leaders/managers	Quantitative	Fidelity of implementation varied between the service delivery settings
i17. De ... (2020)	Belgium	To evaluate the IPS model throughout Belgium	Document analysis/none stated	Qualitative	The perceived facilitators in IPS were related to guidelines and key principles, while the main barriers were lack of lack of funding and lack of communication between stakeholders
i18. Gowdy ... (2003)	USA	To uncover the factors that contributed to differences in competitive employment rates for adults with severe mental illness between high and low-performing programs	Agency leaders/managers, staff	Qualitative	The administrator’s may shape the organizational culture to facilitate the implementation of evidence-based structures and practices
i19. Hamilton ... (2013)	USA	To study a quality improvement approach for implementing evidence-based employment services at specialty mental health clinics	Agency leaders/managers, staff	Mixed	A quality improvement approach resulted in superior patient-level outcomes and improved clinician knowledge, attitudes, and behaviors, in the context of substantial variation among sites
i20. Hasson ... (2011)	Sweden	To identify initial barriers influencing the implementation of supported employment (SE)	Other external stakeholders, agency leaders/managers, staff	Qualitative	Existing regulations for social insurance and employment regulations were perceived as major obstacles to implementation
i21. Hilarión ... (2020)	Spain	To describe the adoption of Individual Placement and Support (IPS) supported employment between 2013 and 2017 in Catalonia (Spain)	Document analysis/none stated	Mixed	Several areas of improvement were described, including the vision of recovery, collaborations between vocational and mental health services, work patterns of practitioners, and views of work as an important treatment
i22. Hillborg ... (2021)	Sweden	To explore the IPS implementation process in a first-episode psychosis (FEP) mental health service team in Sweden	Staff	Qualitative	Integrated process may be achieved by team members who originated from two diverse welfare organizations
i23. Hutchinson ... (2018)	England	To examine whether the implementation was addressing the particular circumstances encountered in each of the sites	Other external stakeholders, agency leaders/managers, staff	Mixed	Maintaining the funding for the Individual Placement and Support services beyond the project period proved to be problematic for many sites
i24. Isett (2007) ...	USA	To analyze implementation issues related to several evidence-based practices for adults with serious mental illness that were included in a national demonstration project	Political/administrative decision-makers, other external stakeholders, agency leaders/managers, staff	Qualitative	The quality of implementation was associated with s are related to these critical areas: financing and regulations, leadership, and training and quality appraisal
i25. Johnson-Kwochka ... (2017)	USA	To evaluate the national prevalence and quality of IPS programs	Political/administrative decision-makers	Qualitative	In the USA, most states provide IPS programs, but the within-state penetration rate and quality of implementation vary widely
i26. Knaeps ... (2012)	Belgium	To measure the possibilities of implementing IPS in Flanders,	Document analysis/none stated	Mixed	The main barriers that impede successful collaboration between vocational rehabilitation services and mental health agencies are the difficult collaboration between governmental agencies for unemployment services and other services, and different values and perceptions between mental health teams and vocational rehabilitation counselors
i27. Latimer ... (2020)	Canada	To evaluate the challenges and strategies encountered in the first 18 months of the At Work program’s implementation, as commissioned by CMHA Toronto and conducted by the Douglas Institute Research Centre	Agency leaders/managers, staff	Qualitative	The national program structure facilitators were flexible eligibility criteria and flexibility in the use of subsidy funds and provision of training support
i28. Lockett ... (2018)	New Zealand	To identify whether, and how, the availability of evidence-based vocational rehabilitation is linked to government policy	Document analysis/none stated	Qualitative	Per policy document analysis, whilst policy reform has commenced, it has not translated into the implementation of IPS widely
i29. Marshall ... (2008)	USA	To report on the factors identified through qualitative analysis that significantly influenced the implementation of evidence-based supported employment	Agency leaders/managers	Qualitative	Three factors, leadership, mastery, and attitudes, were identified as strongly influencing the implementation
i30. Menear ... (2011)	Canada	To shed light on organizational and contextual factors influencing SE implementation in three Canadian provinces	Researchers, political/administrative decision-makers, support organizations, agency leaders/managers, staff	Qualitative	Agencies’ exposure to different institutional pressures, their interactions, and their relationships with other groups and organizations, as well as their values, beliefs, and ideologies, played determining roles in shaping the evolution of SE services
i31. Moe ... (2021)	Norway	To explore the experiences of the front-line workers, known as employment specialists, in the early implementation phase	Staff	Qualitative	Implementing IPS requires adjustments in multiple organizations and can be challenging for employment specialists due to changes in roles and responsibilities
i32. Moe ... (2022)	Norway	To explore the experiences of the front-line workers, known as employment specialists, in the early implementation phase	Researchers, political/administrative decision-makers, other external stakeholders	Qualitative	The process leading to implementing IPS included seeking a way to meet unmet need in work and mental health practice, gathering knowledge and national evidence, and embedding IPS into routine practice
i33. Noel ... (2017)	US	To identify the perceived barriers and facilitators to the sustainment of an evidence-based supported employment program, Individual Placement and Support (IPS)	Agency leaders/managers	Mixed	Funding, prioritization, and workforce characteristics were found key facilitators and barriers to sustainment
i34. Noel ... (2018)	USA	To evaluate the potential of IPS for youth with developmental and/or psychiatric disabilities	Document analysis/none stated	Mixed	A lack of collaboration between systems, competing expectations, and stigma were the main implementation barriers
i35. Oldman ... (2005)	Canada	To describe the transformation of a sheltered workshop program to a program that provides evidence-based supported employment services	Document analysis/none stated	Mixed	The role of agency leadership is important in planning and commitment to quality improvement in implementing change
i36. Parletta ... (2016)	Australia	To compare the financial viability of two approaches (pre-IPS and IPS enhanced) to supported employment	Document analysis/none stated	Quantitative	The government policy towards results-based funding may increase the adoption of IPS practices
i37. Patel ... (2018)	USA	To describe the development and evaluation of e-learning modules as one strategy among a multi-faceted approach to the implementation of individual placement and support (IPS),	Agency leaders/managers, staff	Quantitative	Feedback collected from the training program may inform the design of subsequent training programs
i38. Pogoda ... (2011)	USA	To document perceived barriers to supported employment implementation as described by Department of Veterans Affairs (VA) employees	Agency leaders/managers, Staff	Qualitative	Employees’ paternalistic attitudes about individuals with serious mental illness were reported as barriers to implementation
i39. Pogue ... (2021)	USA	To examine the growth of IPS in the United States from 2016 to 2019, comparing growth rates for stateswithin and outside the learning community	Political /administrative decision-makers	Qualitative	Participating in the IPS Learning Community may foster penetration and sustainment of high-fidelity IPS
i40. Priest ... (2020)	New Zealand	To study participation in a newly established IPS program	Agency leaders/managers	Mixed	Adoption is dependent on national supports, financial resources, and agency leadership commitment
i41. Rapp ... (2010)	USA	To report barriers to EBP implementation in one state that sought to implement supported employment and integrated dual diagnosis treatment	Agency leaders/managers, staff	Qualitative	The behavior of supervisors, front-line staff, and other professionals in the agency were barriers to implementation
i42. Roeg ... (2020)	Netherlands	To explore IPS model fidelity and employment outcomes in supported housing services and mental health treatment services	Support organization	Qualitative	Organizational and financial structures affect the quality of implementation
i43. Salkever ... (2018)	USA	To study (1) the influence of client characteristics on take-up probability and (2) the possible impacts	Document analysis/none stated	Quantitative	State initiatives, clients’ diagnoses, prior work history, health and demographic characteristics, and geographic accessibility may affect the reach of the model
i44. Schneider ... (2012)	England	To describe the implementation of individual placement and support (IPS)	Document analysis/none stated	Qualitative	Systematic approach to implementation may increase the success of the adoption of the model
i45. Sharek ... (2022)	Ireland	To explore how IPS Employment Specialists (ES) and Occupational Therapist (OT) Managers integrated and embedded IPS within traditionally oriented MDTs as part of a national reform program	Agency leaders/managers, staff	Qualitative	Contexts, strategies, and attitudes towards the target group affect the implementation of IPS
i46. Stirling ... (2018)	Australia	To explore why the model is not yet widely available	Document analysis/none stated	Qualitative	Consistent measures, change indicators, direction, and time frames were lacking in policy and strategy documentation
i47. Swain ... (2010)	USA	To discern the number of sites that sustained practices 2 years after implementation	Support organization, Agency leaders/managers	Mixed	Financing, training, fidelity, and agency leadership separated sustaining sites from non-sustaining sites
i48. Swanson ... (2014)	USA	To examine how 3 state implementation teams helped separate agencies to partner on IPS-supported employment	Agency leaders/managers, staff	Qualitative	Leaders used several strategies to promote implementation
i49. Talbot ... (2018)	England	to use it to guide a description of IPS implementation based on observations, spanning 6 months in community forensic mental health settings	Document analysis/none stated	Qualitative	Implementation of individual placement and support in forensic mental health settings is complex and requires robust planning and collaboration with internal and external agencies
i50. Thomas ... (2009)	USA	To discuss the implementation and ongoing development of evidence-based supported employment services	Document analysis/none stated	Qualitative	Implementation of IPS is facilitated by the practitioners’ paradigm shift in providing service and the need for funding streams supporting evidence-based, recovery-oriented employment services
i51. van Duin ... (2013)	Netherlands	To examine the large-scale implementation of the National Multidisciplinary Guideline for Schizophrenia in the Netherlands	Staff	Quantitative	Participation in Quality Improvement Collaboration improved professional performance
i52. van Duin ... (2021)	Netherlands	To examine methods used to implement interventions, barriers and facilitators, and implementation outcomes (fidelity, uptake, and availability)	Staff	Mixed	Implementing IPS benefits from a structure approach to implementation supports
i53. van Erp ... (2007)	Netherlands	To assess fidelity, employment outcomes, and facilitators of and barriers to successful implementation	Support organization, agency leaders/managers, staff	Mixed	Important facilitators are regular meetings of stakeholders in mental health care and vocational rehabilitation, stakeholders’ experienced ownership of IPS and collaboration, the mandate and influence of the decision makers involved, and secured IPS funding
i54. van Weeghel ... (2020)	Netherlands	To discuss the rise of individual placement and support (IPS) within vocational services for people with severe mental illness (SMI)	Document analysis/none stated	Qualitative	An implementation study and a multisite randomized controlled trial have indicated that IPS is feasible and effective in the Netherlands, and the number of enrolled IPS participants doubled between 2016 and 2017, largely due to national funding
i55. Vukadin ... (2018)	Netherlands	To explore facilitators and barriers with regard to the organizational and financial implementation strategy for IPS	Political/administrative decision-makers, other external stakeholders, agency leaders/managers, staff	Qualitative	The key principles of the IPS model, stakeholder meetings, experienced ownership and collaboration, mandate and influence of decision-makers, and secured funding were perceived as facilitators for IPS, while the experienced rigidity of the IPS model fidelity scale, lack of independent fidelity reviewers, temporary and fragmented funding, lack of communication between decision-makers and practitioners, and negative attitudes among mental health clinicians were perceived as barriers
i56. Vukadin ... (2021)	Netherlands	To explore experiences with Individual Placement and Support using a multifaceted implementation strategy	Staff	Qualitative	Organizational barriers to IPS execution, financial barriers to IPS execution, and experiences with the pay-for-performance element affect the implementation
i57. Waghorn ... (2007)	Australia	To describe the implementation issues encountered at seven sites pioneering evidence-based employment services	Agency leaders/managers, staff	Qualitative	The major difficulties were related to service integration and utilizing the existing federal disability employment system
i58. Waghorn ... (2020)	Australia	To summarize the major developments in Australia since the first introduction of Individual Placement and Support (IPS) in 2005	Document analysis/none stated	Mixed	Promising implementation is constrained in the adult community mental health sector by factors including low priority for rehabilitation in the public mental health system
i59. Zhen‐Duan ... (2022)	US	To explore how stakeholders responded to research evidence regarding supported employment	Political/administrative decision-makers	Qualitative	Supportive leaders, legislation, memoranda of understanding, and cooperative agreements were crucial to acquiring resources and successfully implementing SE programs

^*^The table holds references to the Additional file 2

**Fig. 2:**
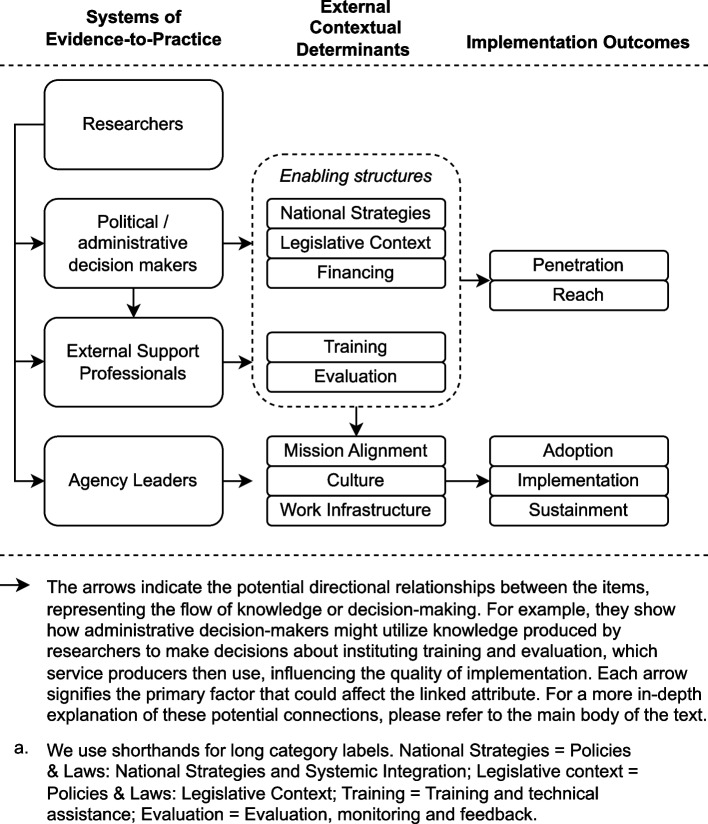
Associations between the systems of evidence-to-practice, external contextual determinants, and implementation outcomes: a conceptual model

Table 2 displays the characteristics of evidence sources, presenting the frequency of observations and the sources by thematic categories. Table 2 also shows the frequencies of observations for each determinant cross-tabulated by each implementation outcome. Implementation stood out as the dominant outcome, covered in 46 articles, while adoption and penetration/reach were discussed in 29 and 26 of the articles, respectively. Sustainment found the least attention (*n* = 16). The data prominently showcased local determinants, including work infrastructure (*n* = 34), mission alignment (*n* = 33), and culture (*n* = 29), emphasizing their significance in the adoption and implementation phases. Of these, mission alignment was highly prevalent in sustainment articles. The concept of agency leaders was discussed in 38 articles, and their role was highly present in adoption, implementation, and sustainment articles. Researchers (*n* = 11) and political/administrative decision-makers (*n* = 25) were most frequently cited in articles concerning sustainment and penetration/reach. They had in common their frequent association with national strategies (*n* = 25), while the former was more often associated with evaluation, monitoring, and feedback (*n* = 25) and the latter with financing (*n* = 41). Discussion on sustainment and penetration/reach also frequently associated with national strategies, legislative context (*n* = 31), and financing. External support professionals (*n* = 20) were relatively highly represented in the articles on sustainment. Figure 2 is a diagram that depicts the relative positions of categories and directions of influence between them in a conceptual model.

**Table 2:** Frequency of observations and sources of evidence: frequencies presented in total and divided into facilitators and barriers by implementation outcome

Concept	Adoption	Implementation	Sustainment	Penetration/reach	Total, *N* (%)
Total, *N* (%)	29 (49.2%)	46 (78.0%)	16 (27.1%)	26 (44.1%)	59 (100%)
Policies and laws: national strategies and systemic integration	Facilitators, *n* = 15 (51.7%^a^), barriers, *n* = 14 (48.3%): i4^*^, i6, i10, i11, i19, i23, i24, i27, i30, i31, i40, i44, i45, i49, i58	Facilitators, *n* = 17 (37.0%), barriers,* n* = 18 (39.1%): i4, i6, i10, i11, i14, i23, i24, i27, i28, i30, i31, i32, i33, i44, i45, i49, i55, i58	Facilitators, *n* = 9 (56.2%), barriers, *n* = 9 (56.2%): i4, i6, i9, i14, i23, i30, i33, i44, i55, i58	Facilitators, *n* = 15 (57.7%), barriers, *n* = 13 (50.0%): i4, i9, i10, i24, i28, i30, i32, i33, i39, i44, i45, i46, i54, i55, i58, i59	25 (42.4%)
Policies and laws: legislative context	Facilitators, *n* = 16 (55.2%), barriers, *n* = 16 (55.2%): i1, i2, i4, i6, i10, i11, i15, i27, i30, i31, i44, i45, i48, i49, i57, i58	Facilitators, *n* = 24 (52.2%), barriers, *n* = 28 (60.9%): i1, i2, i4, i5, i6, i10, i11, i13, i14, i15, i16, i17, i20, i21, i27, i28, i30, i31, i32, i42, i44, i45, i48, i49, i55, i56, i57, i58	Facilitators, *n* = 10 (62.5%), barriers, *n* = 11 (68.8%): i2, i4, i6, i14, i15, i21, i30, i44, i55, i57, i58	Facilitators, *n* = 17 (65.4%), barriers, *n* = 18 (69.2%): i2, i4, i5, i10, i21, i25, i28, i30, i32, i42, i43, i44, i45, i48, i55, i57, i58, i59	31 (52.5%)
Financing	Facilitators, *n* = 22 (75.9%), barriers, *n* = 20 (69.0%): i2, i4, i6, i8, i10, i11, i15, i19, i24, i27, i29, i30, i35, i38, i40, i44, i47, i48, i49, i50, i57, i58	Facilitators, *n* = 29 (63.0%), barriers, *n* = 31 (67.4%): i2, i4, i6, i7, i8, i10, i11, i12, i15, i17, i21, i24, i26, i27, i28, i29, i30, i32, i33, i38, i42, i44, i48, i49, i50, i52, i53, i55, i56, i57, i58	Facilitators, *n* = 14 (87.5%), barriers,* n* = 13 (81.2%): i2, i4, i6, i7, i9, i15, i21, i30, i33, i44, i47, i55, i57, i58	Facilitators, *n* = 20 (76.9%), barriers, *n* = 17 (65.4%): i2, i3, i4, i9, i10, i21, i24, i28, i30, i32, i33, i36, i39, i42, i44, i48, i54, i55, i57, i58, i59	41 (69.5%)
Training and technical assistance	Facilitators, *n* = 19 (65.5%), barriers, *n* = 17 (58.6%): i2, i4, i10, i15, i19, i27, i29, i30, i31, i35, i38, i41, i44, i45, i48, i49, i50, i51, i58	Facilitators, *n* = 22 (47.8%), barriers, *n* = 21 (45.7%): i2, i4, i7, i10, i15, i21, i27, i29, i30, i31, i34, i37, i38, i41, i44, i45, i48, i49, i50, i51, i52, i53, i58	Facilitators, *n* = 9 (56.2%), barriers, *n* = 8 (50.0%): i2, i4, i7, i9, i15, i21, i30, i44, i58	Facilitators, *n* = 11 (42.3%), barriers, *n* = 9 (34.6%): i2, i4, i9, i10, i21, i30, i39, i44, i45, i48, i58	27 (45.8%)
Evaluation, monitoring and feedback	Facilitators, *n* = 17 (58.6%), barriers, *n* = 16 (55.2%): i1, i2, i4, i6, i8, i10, i23, i30, i31, i40, i41, i44, i45, i48, i49, i50, i58	Facilitators, *n* = 21 (45.7%), barriers, *n* = 21 (45.7%): i1, i2, i4, i6, i7, i8, i10, i18, i21, i23, i30, i31, i41, i44, i45, i48, i49, i50, i52, i53, i58	Facilitators, *n* = 9 (56.2%), barriers, *n* = 9 (56.2%): i2, i4, i6, i7, i21, i23, i30, i44, i58	Facilitators, *n* = 12 (46.2%), barriers, *n* = 11 (42.3%): i2, i4, i10, i21, i25, i30, i39, i44, i45, i48, i58, i59	25 (42.4%)
Mission alignment	Facilitators, *n* = 23 (79.3%), barriers, *n* = 22 (75.9%): i1, i2, i4, i6, i8, i10, i11, i15, i23, i27, i30, i31, i35, i38, i41, i44, i45, i47, i48, i49, i50, i57, i58	Facilitators, *n* = 28 (60.9%), barriers, *n* = 31 (67.4%): i1, i2, i4, i5, i6, i7, i8, i10, i11, i12, i14, i15, i18, i20, i21, i23, i27, i30, i31, i33, i38, i41, i44, i45, i48, i49, i50, i53, i55, i57, i58	Facilitators, *n* = 14 (87.5%), barriers, *n* = 15 (93.8%): i2, i4, i6, i7, i14, i15, i21, i23, i30, i33, i44, i47, i55, i57, i58	Facilitators, *n* = 12 (46.2%), barriers, *n* = 13 (50.0%): i2, i4, i5, i10, i21, i30, i33, i44, i45, i48, i55, i57, i58	33 (55.9%)
Culture	Facilitators, *n* = 18 (62.1%), barriers, *n* = 18 (62.1%): i1, i6, i8, i10, i11, i15, i19, i22, i23, i27, i29, i30, i31, i38, i41, i45, i49, i58	Facilitators, *n* = 23 (50.0%), barriers, *n* = 28 (60.9%): i1, i5, i6, i7, i8, i10, i11, i12, i14, i15, i17, i18, i20, i22, i23, i27, i29, i30, i31, i34, i38, i41, i45, i49, i52, i53, i55, i58	Facilitators, *n* = 7 (43.8%), barriers, *n* = 8 (50.0%): i6, i7, i14, i15, i23, i30, i55, i58	Facilitators, *n* = 5 (19.2%), barriers, *n* = 6 (23.1%): i5, i10, i30, i45, i55, i58	29 (49.2%)
Work infrastructure	Facilitators, *n* = 21 (72.4%), barriers, *n* = 20 (69.0%): i1, i2, i6, i8, i11, i15, i19, i22, i23, i27, i29, i30, i31, i35, i38, i41, i44, i48, i49, i57, i58	Facilitators, *n* = 28 (60.9%), barriers, *n* = 32 (69.6%): i1, i2, i5, i6, i7, i8, i11, i13, i14, i15, i16, i20, i22, i23, i27, i28, i29, i30, i31, i33, i34, i38, i41, i42, i44, i48, i49, i53, i55, i56, i57, i58	Facilitators, *n* = 11 (68.8%), barriers, *n* = 12 (75.0%): i2, i6, i7, i14, i15, i23, i30, i33, i44, i55, i57, i58	Facilitators, *n* = 10 (38.5%), barriers, *n* = 11 (42.3%): i2, i5, i28, i30, i33, i42, i44, i48, i55, i57, i58	34 (57.6%)
Researchers	Facilitators, *n* = 7 (24.1%), barriers, *n* = 7 (24.1%): i2, i4, i30, i44, i49, i50, i58	Facilitators, *n* = 9 (19.6%), barriers, *n* = 9 (19.6%): i2, i4, i30, i32, i33, i44, i49, i50, i58	Facilitators, *n* = 6 (37.5%), barriers, *n* = 6 (37.5%): i2, i4, i30, i33, i44, i58	Facilitators, *n* = 9 (34.6%), barriers, *n* = 8 (30.8%): i2, i4, i25, i30, i32, i33, i39, i44, i58	11 (18.6%)
Political/administrative decision-makers	Facilitators, *n* = 13 (44.8%), barriers, *n* = 13 (44.8%): i2, i4, i6, i10, i11, i23, i24, i29, i30, i38, i48, i49, i58	Facilitators, *n* = 18 (39.1%), barriers, *n* = 20 (43.5%): i2, i4, i6, i7, i10, i11, i17, i23, i24, i26, i28, i29, i30, i32, i33, i38, i48, i49, i55, i58	Facilitators, *n* = 10 (62.5%), barriers, *n* = 9 (56.2%): i2, i4, i6, i7, i9, i23, i30, i33, i55, i58	Facilitators, *n* = 16 (61.5%), barriers, *n* = 13 (50.0%): i2, i4, i9, i10, i24, i25, i28, i30, i32, i33, i39, i48, i54, i55, i58, i59	25 (42.4%)
External support professionals	Facilitators, *n* = 13 (44.8%), barriers, *n* = 10 (34.5%): i2, i4, i10, i15, i23, i27, i31, i35, i40, i44, i48, i51, i58	Facilitators, *n* = 15 (32.6%), barriers, *n* = 13 (28.3%): i2, i4, i10, i15, i21, i23, i27, i28, i31, i33, i37, i44, i48, i51, i58	Facilitators, *n* = 9 (56.2%), barriers, *n* = 8 (50.0%): i2, i4, i9, i15, i21, i23, i33, i44, i58	Facilitators, *n* = 12 (46.2%), Barriers, *n* = 10 (38.5%): i2, i4, i9, i10, i21, i25, i28, i33, i44, i48, i54, i58	20 (33.9%)
Agency leaders	Facilitators, *n* = 27 (93.1%), barriers, *n* = 25 (86.2%): i1, i2, i4, i6, i8, i10, i11, i15, i19, i23, i24, i27, i29, i30, i31, i35, i38, i40, i41, i44, i45, i47, i48, i49, i50, i57, i58	Facilitators, *n* = 31 (67.4%), barriers, *n* = 33 (71.7%): i1, i2, i4, i5, i6, i7, i8, i10, i11, i12, i14, i15, i18, i21, i23, i24, i27, i29, i30, i31, i33, i38, i41, i44, i45, i48, i49, i50, i52, i53, i55, i57, i58	Facilitators, *n* = 15 (93.8%), barriers, *n* = 15 (93.8%): i2, i4, i6, i7, i9, i14, i15, i21, i23, i30, i33, i44, i47, i55, i57, i58	Facilitators, *n* = 14 (53.8%), barriers, *n* = 14 (53.8%): i2, i4, i5, i9, i10, i21, i24, i30, i33, i44, i45, i48, i55, i57, i58	38 (64.4%)

^a^The proportion of articles holding reference to the denoted determinant-outcome association divided by the total number of articles holding references to the outcome

^*^The table holds references to the Additional file 2. List of references for included studies

### External contextual determinants

#### Policies and laws: national strategies and systemic integration

National or regional strategies were described as promoting the uptake and implementation of IPS [38] and appeared to be backed by administrative decisions about responsibility-sharing or funding. These policies included national mental health strategies [39], guidelines [40], and agreements on implementation support issues [18, 19]. The IPS model was perceived as a contributor to the strategic goal of implementing the recovery approach and serving as a vehicle for producing system reform at national and regional levels [18]. Congruence with other national policy goals and frameworks, such as social inclusion [39] and participation [41], was found to facilitate the incorporation of IPS principles into national mental health care policies. Systematic approaches in providing implementation support could support national strategies [42] whereas a mismatch between overarching national strategies and a lack of programs to implement IPS to achieve the goals of these strategies was reported to lead to lower penetration or adaptation of the IPS model [39, 43, 44].

One feature of the national strategies was the aim of expanding the clientele from persons with severe mental disorders such as psychotic disorders to those with any mental disorder, leading to implementing IPS in various care settings, e.g., forensic or psychiatric housing programs [45, 46]. The implications of different work infrastructures on implementation are discussed in a separate section below (work infrastructure).

#### Policies and laws: legislative context

Legislative contexts concerning mental health and employment were reported to impact the implementation of the IPS model. Laws that mandate employment services for individuals with severe mental illness [47] or policies redirecting services from activities not following the IPS model [48] increased the adoption of IPS programs. On the other hand, the availability of competing practices [49–51], procedures mandated by policies but not supported by research, such as work capacity assessments [52–54] or mandated lengthy referral processes [54], were reportedly at odds with the implementation of IPS with adherence to model guidelines. Social insurance criteria that excluded clients based on expected employment outcomes [55] or received benefit types [56] were also reported as barriers. The policy of allocating decision-making and management of services to local authorities was reported to hinder adoption due to low prioritization at the local level [41]. Laws and regulations related to sharing client information and access to data and mandated use of multiple information systems were reported to complicate the implementation of IPS [44, 57, 58]. Legally mandated limitations on using data could be circumvented by strategic actions by the administrative authorities or local leaders [18, 44].

#### Financing

The availability of funding was critical for adopting and implementing IPS across the settings. National or regional development projects were often used in the adoption phase [59, 60]. Sustained direct funding schemes through health ministries or other governmental organizations were used to increase the use or adoption within the service system or provide the necessary flexibility to implement the model as intended at the local level [38, 48, 60]. A state-level funding mechanism was associated with statewide uptake of the model [49]. Payments based on achieved results were reported to facilitate sustained implementation [49, 61]. Many studies reported that a well-managed transition from projects to sustained programs was a critical period.

Specific funding mechanisms were reported as barriers to the successful implementation of IPS. Payment models that were based on specific medical diagnoses rather than outcomes [62, 63] and separate or divided sources of funding [18, 41, 42, 51] hindered the implementation. Set or predefined funding duration to funding [41, 46, 57, 59, 64], restrictions on financing employment services as health services [51, 65], and rules that penalize short employment contracts [51] were also perceived to impact the quality of implementation negatively. Funding contracts covering a broader set of programs could include criteria conflicting with the IPS fidelity criteria [50, 63].

#### Training and technical assistance

Training and technical assistance were reported to facilitate the implementation of IPS. Sources for training and assistance included support from national, state, and regional organizations [66, 67] and IPS/EBP development projects [42, 68], as well as openly available guidelines and training material provided by the program’s developers [55]. These supports reportedly helped those putting the model into practice with goal setting and providing a sense of purpose [44, 50], helped providers to work systematically according to protocols and improved their knowledge of evidence-based practices [44, 68], and provided opportunities to share knowledge and experiences with other sites [55]. Agency leaders [44] and staff [62, 69] were reported to benefit from initial training and assistance [65].

#### Evaluation, monitoring, and feedback

National, state, and regional organizations [19, 42, 67] and outside experts were used to conduct evaluations and monitoring of the implementation of IPS that were often reported in conjunction with training and technical assistance. Routinely assessing implementation was perceived to help ensure that the model is implemented as intended over time [62], and imposing continuous evaluation by agency leaders may increase the probability of the sustainment of the program [70]. In some cases, fidelity above a certain threshold was used as a prerequisite for funding by national or regional decision-making organizations [71]. Disseminating the results on the effectiveness of IPS reportedly increased the model's adoption [18], and evaluations and monitoring were used to motivate leaders to maintain or reinstate high-fidelity services [40]. In contrast, the lack of results from monitoring or evaluations could discourage agency leaders from following national guidelines that promoted the use of IPS [46].

### Local factors affected by external context

#### Mission alignment

Both the recovery approach [41, 59, 63, 72] and evidence-based policy commitment [69, 73] were reported to facilitate the reorienting of organizational goals to be consistent with IPS implementation and sustainment. The shift in organizational goals was associated with the de-implementation of vocational services that lacked evidence-based support and were supported by structural changes and financial arrangements through administrative decisions [74].

The non-alignment with organizational goals was reported to hinder the model’s implementation. The model could be at odds with existing organizational goals based on traditional medical or vocational services [75, 76]. These goals could be mandated by existing rules and regulations [53]. Challenges were reported when collaborating partners from different organizations had different goals in their respective organizations [52, 53, 60, 77], which could lead to giving lower priority to collaborating with the IPS team [52, 70].

#### Culture

Acceptance of the model by the professionals, professional norms, and local attitudes was reported as important for the uptake and implementation of the model. Understanding the program logic [60, 73] and recognizing unmet user needs [72, 78] were associated with the changes in acceptance of the model and professional norms. Several studies found that influencing practitioners’ professional norms and attitudes was an important goal during the adoption period. During this time, practitioners could learn about the rights and needs of users, the benefits of IPS, and community resources; changes in these attitudes would lead to better implementation results [55, 58, 72, 79]. Receiving training and support from site managers and national organizations [65], experiencing bringing together service functions as intended, and sharing success stories [58] were perceived to facilitate the implementation and sustainment of the model.

In several studies, the practitioners were reported to view IPS as conflicting with the core beliefs or principles of care. The practitioners may see employment or financial self-sufficiency as a not crucial outcome for health services [49, 70, 77], or they may see IPS as an inferior or unnecessary service [46, 63, 77]. Negative attitudes about the capabilities of the target group could lead to lower referrals to IPS [41, 44, 57, 65, 80], referrals to employment services not supported by research [48, 55, 57], exclusion from the service [55, 80], inadequately bringing together service functions [48, 65], and poor collaboration with external partners [53, 63].

#### Work infrastructure

IPS was implemented within mental health services, outside of mental health services, or as a collaboration between different organizations. Programs in community mental health settings rated higher fidelity than those in rehabilitation centers, housing units, or independent programs [45, 81, 82]. Providing the service in a mental health care setting was reported to lead to higher and shorter referral processes. Transforming a work setting to a high-fidelity IPS service was reported to require creating or protecting designated or reserved staff roles, adjusting the number of clients assigned to a single professional, or renegotiating the existing job descriptions [62, 69, 74]. The infrastructure related to continuous support was reported to promote the model’s sustainment [70].

In the situations where multiple organizations implemented the model together, strategies and agreements on financial matters [54, 83], identifying shared clients [83], and practical arrangements such as office space [83] and designated contact persons [64] were reported to facilitate implementation. The willingness to share expertise and the complementary experiences of different stakeholders [46] can also help with implementation. On the other hand, organizations that are expected to collaborate may resort to conflicting service processes [52, 53, 56, 60]. In situations where multiple organizations worked together, the absence of formal agreements led to poor referrals [54] and hindered effective implementation [41].

### Systems of evidence-to-practice

#### Researchers

Researchers’ active involvement in developing and implementing strategies for disseminating the IPS model included collaborating directly with political and administrative decision-making, national and regional support organizations, and the implementing agencies. In the USA, the promotion of the decision-makers’ participation in the learning community was found to encourage interagency collaboration at the state level [47], including arrangements for state-level funding [66, 84] and evaluation and training support [47, 84], resulting in a higher number of IPS programs per state population and faster growth in penetration [84]. In Australia, national-level advocacy included a group of researchers promoting the IPS model to state and federal politicians and government department administrators, leading to decisions related to funding and development projects [40].

The US Learning Collaborative, a researcher-led initiative for disseminating the IPS model, has also produced numerous research collaborations supporting the model’s spread across the settings [19]. These collaborations were found in the form of partnering with the developers of the model to produce new evidence or support implementation [18, 40, 51, 59], training experts at the national level [40], or collaborating with those putting the model into practice directly [48, 51].

#### Political/administrative decision-makers

The model’s penetration was facilitated by decisions by the state politicians and administration [18, 40, 48, 55] or local political decision-makers [77, 85]. The dedication and enthusiasm of actors at the administration level were reported to facilitate the necessary collaborations [41, 48, 49, 62]. Enthusiastic state IPS coordinators and administrative authorities were reported to foster a culture shift in agencies, leading to high-fidelity implementation and sustained model use [49, 71].

Political/administrative decision-making was reported to induce changes in national policies. Recurrent funding decisions [48] and funding designated for IPS were reported as a facilitator of local implementation and service system penetration [49]. Also, decisions to change policy regulations and protocols, rules for referring to services, and providing support resources for implementing the model were reported to support the system-level adoption of the model [18, 49]. Enforcing national strategies and guidelines was reported to stipulate political or administrative decision-making at the local level [38]. Administrative collaboration, including coordination, consensus-building, or formal agreements between responsible agencies [18, 51, 54] or professional networks [83], reportedly facilitated coordinated referral processes and joint data collection efforts, resulting in higher penetration [49, 50] and quality of services at the aggregate level [18].

Administrators’ commitment to models not supported by research [49] and the lack of state-level collaboration between administrators in different agencies were associated with non-aligned strategies for employment services for the target group [47]. Studies also reported the ambiguity that decision-makers face when facing different potential service models [41, 70] and when considering increasing the penetration outside the specialized mental health care system [41]. One study reported ambiguity in that the strategies might recognize the significance of enhancing employment rates for individuals with mental disorders but consistent implementation plans were lacking [39]. In addition, administrative hesitancy was linked to the lack of power in decision-making [70, 83].

#### External support professionals

National or regional supporting organizations were reported to promote collaboration, funding, training, and evaluation. Their form varied from organizations created to support individual IPS projects [18, 19, 46, 67] to quality improvement collaborations involving several EBPs [42, 68] and contracting support services from other sites that implement the service [18]. These collaborations often included partnerships with and resources from university researchers [18, 66]. Implementing these supports could be a feature of a dissemination plan [49], and the number of active IPS programs was associated with the number of national trainers [19].

Support organizations could help the implementing sites to create implementation strategies [65, 83], budget plans involving one or more agencies [62], and encourage agency leaders to proceed with the implementation in problematic situations [83]. Training, technical assistance, fidelity, and outcome monitoring were often reported as critical aspects of implementation support [18, 46, 49, 83, 86]. Evaluation and monitoring data were reported to have been used to increase accountability and motivate decision-makers to increase funding [18, 49]. Centralized enforcement of adherence to model guidelines and outcome monitoring was found to improve the quality of implementation over time across sites [49]. Fidelity and outcome monitoring also reportedly facilitated both national consensus-building and supervision based on achieved results at the local level [18].

Poor or lack of national implementation support was reported to lead to fewer links and communications between academics and implementing agencies and low leadership involvement [44]. Removal of regional leadership and a decline in national/regional training and evaluation supports were found to lead to lower quality implementation of once-sustained programs [40]. Short timeframes for national development projects that provided external support for local sites were associated with challenges in achieving organizational structural changes in the service-producing organizations [46, 68].

#### Agency leaders

Senior leaders, often motivated by the recovery approach and the evidence base [48, 59, 63], were the actors who promoted ‘systemic transformation’ [51] and placed the IPS within a broader area of strategy for psychosocial services provided by the care organization [72]. Committed senior leaders communicated the importance of the recovery approach and services tailored to each person’s specific needs, which was reported to lead to higher quality services [62, 71, 87]. Prioritizing and enforcing strategies and actions, often using the steering group, was decisive as it affected several aspects of the effort, including the affecting organizational policy, promoting the program’s credibility among the professionals, the methods for cooperation, and the financing decisions [51, 55, 62, 63, 70, 71, 83]. Senior leaders’ commitment to the guidelines to ensure the IPS program is being implemented as intended [40, 54] and enforcing fidelity monitoring [40, 70] were reported to facilitate sustained implementation. In the situations where multiple organizations worked together, combining leadership outside the provider organization was perceived beneficial for implementation [59].

Agency senior leaders’ failure to align the IPS model principles with organizational goals and inadequate agency prioritization [44, 65, 71, 77] led to poorer implementation or non-sustainment. Lack of enthusiasm and promotion of the model [70, 71], not being able to channel funding [65, 77], and not using performance-related indicators [46] can also hinder its implementation or sustainment. Failure of steering groups to commit or their dissolution after the project period was reported to cause a cessation of funding or poor coordination with external partners [56, 77].

## Discussion

### Summary of evidence

We investigated and identified the external contextual factors that can influence the adoption, implementation, sustainment, and scale-up of the Individual Placement and Support (IPS) model, an evidence-based practice (EBP) for acquiring employment for persons with mental disorders. In this scoping review, we found that policies and laws, financing, and administratively instituted support resources were consistently conceived as facilitators or barriers to implementation across diverse settings in Western countries. Organizational mission alignment, culture, and work infrastructure were also identified as externally influenced factors that facilitated or hindered the implementation. Furthermore, we found these determinants of local implementation to be affected by strategies and actions of researchers, political and administrative decision-makers, external support professionals, and the implementing organization’s leaders support. Collectively, these actors formed and participated in complex nationally or regionally varying constellations facilitating or hindering the local implementation effort and model’s penetration into service systems.

To study how external contextual factors can affect implementation processes at the local level [4, 5, 88], we used CFIR framework [4, 33] categories as the starting point of our data analysis. It allowed us to classify the data coherently to a variable degree depending on the variables, and the framework-to-data fit may be considered moderate. Our results indicate several areas where the CFIR framework did not give the best attainable framework-to-data match. First, we introduced ‘evaluation and monitoring’ and ‘training and technical assistance’ as external context categories. These items could have been coded as enactments of ‘national strategies’ or omitted from this analysis by categorizing them under CFIR’s implementation process domain. Given the high prevalence of these items in the data and their relative position to other concepts, including these items as external contextual provided an improved framework-to-data match. We also considered including these items feasible, as facilitation of implementation is considered a central or important feature in other implementation frameworks [36, 89]. Second, we refined the CFIR categorization by distinguishing between ‘national strategies and systemic integration’ and ‘legislative context’ as separate subcategories within the ‘policies and laws’ category. This decision does not represent a deviation from CFIR but acknowledges the qualitative difference between the items, supported by the prevalence in data and distinctiveness of the items. A comparable distinction has been made in other frameworks [5, 89]. The legislative context category included observations on many broader structural policy arrangements outside the healthcare administration’s decision-making power, which may be important when considering strategies for the model expansion in real-world settings.

The most significant deviation from CFIR was how external stakeholders were considered. The analytical choice in this study was to perceive their role through the lens of socio-ecological systems [7, 35, 36], which allows the incorporation of the agency of different stakeholders. The most significant difference to CFIR was to include observations describing the stakeholders’ strategies and actions that were directed not solely towards local implementation efforts but also towards affecting the other external contextual determinants and scaling up efforts [29, 30]. The proposed ‘systems of evidence-to-practice’ category holds findings that could be considered in CFIR’s ‘partnerships and connections’ category, ‘implementation process domain’, or be excluded from the analysis to the extent they did not directly refer to the external influences on the local organization. Implementation science theorists have called after studies incorporated observations on multi-level strategies [10] or accountability mechanisms reflecting both the organizational and systemic levels [90–92]. With this regard, our classification resulted in improved framework-to-data match and narrowed the gap in knowledge with regard to the real-world processes through which different key stakeholders, namely researchers, political and administrative decision-makers, and support organizations, actively promote the translation of the evidence to actual implementation outcomes.

Our results support considering certain ‘inner setting’ items such as ‘mission alignment’, ‘culture’, and ‘work infrastructure’ as factors influenced by the broader structure of societal norms and arrangements. They were present in data describing the external and organizational contextual features’ interaction and often targets of interventions by the external facilitators. These items represent one facet of the non-distinctiveness of the line between the outer and inner contexts [4]. Similar items were also included in the taxonomy of external context items affecting the implementation of complex evidence-based health interventions by Watson et al. [2]. To conclude, our analysis led to a slightly modified list of CFIR’s ‘outer setting domain’ categories which we labeled as ‘enabling structures’, denoting the core components affecting the implementation and scale-up efforts.

Our analysis suggests several areas for further studies. First, our study confirms a knowledge gap: the external context factors were a systematically under-emphasized area of empirical research [2, 10, 11] and mainly described in an exploratory fashion [2] also within empirical IPS research. Second, the heterogeneity and perceived variability in the quality of the data suggest that deliberate efforts should be directed to establish more stringent operational definitions of external context. Our conceptual model represents a plausible way to organize the complexity of ‘external contextual’ items and stakeholder relationships needed for such research. Future studies would also benefit from the use of clearly defined and operationalized implementation outcomes. Differentiating between outcomes could prompt researchers to concentrate on under-researched areas. The most significant knowledge gap highlighted by our data is the studies dedicated specifically to sustainment outcomes, followed by system-level outcomes. Third, future studies should move the non-systematic approach to socio-ecological systems that partake in translating evidence to practice. Extending the study of the implementation strategies and their implementation [1] to external stakeholders with their respective organizational contextual underpinnings, potentially with the help of organizational theories [10, 93], could improve the findings’ completeness and real-world relevance. Finally, our results highlight leadership’s moderating role in context-implementation relationships not only when considering local organizational contexts [94, 95] but also when accounting for external contextual influences, marking it as a crucial topic for future studies.

### Strengths and limitations

Our study has several strengths, including being strictly based on studies reporting empirical insights concerning the actual implementation and excluding theoretical or descriptive accounts, the large number of reviewed articles, the focus on a single well-defined intervention, and the heterogeneity of the organizational and societal settings from which our results were derived. However, there are several limitations to consider. First, the generalizability of our findings outside IPS implementation may be limited. This is because different health sectors, organizational settings, and EBPs each have unique characteristics that may require distinct implementation frameworks and models [96]. Second, the generalizability is bounded by all included studies being conducted in rich developed countries. Third, our results may be subject to author bias as a considerable portion of the reviewed literature was written by researchers associated with the model’s creation and early expansion. As an abductive thematic analysis, the results of this study may be biased by the authors’ judgments. Also, coding for thematic analysis was solely conducted by the first author, potentially impacting the reliability and validity of the results. Fourth, in line with our protocol and PRISMA-ScR guidelines, we did not systematically assess the quality of the included studies. We acknowledge, however, that such an assessment would have enhanced the reliability of our results, given the perceived variance in the quality of the data analyzed. In this study, we have aimed to reduce these biases by following a standardized reporting protocol for scoping reviews [25] and being explicit about the analytical choices.

## Conclusions

Our scoping review provides an empirically based perspective for discussing the role of the external contextual factors affecting EBP implementation and scale-up. Our study summarises empirical research that reports structural, policy, and legal levels and support systems as facilitators or barriers to the implementation effort. Our findings highlight the importance of different stakeholders’ unique characteristics and collaboration at different socio-ecological system levels. The results indicate gaps in knowledge in implementation science and offer suggestions for future research.

### Supplementary Information

**Additional file1. **PRISMA-ScrR Checklist.

**Additional file 2.** List of references for included studies.

**Additional file 3.** List of references for excluded full-text studies.

## Data Availability

The dataset used to generate the tables in the current study is available in the Open Science Framework repository [97].

## Abbreviations

CFIR: Consolidated Framework for Implementation Research
EBP: Evidence-based practice
IPS: Individual Placement and Support
JBI: Joanna Briggs Institute
PCC: Population/Concept/Context framework
PRISMA-ScR: Preferred Reporting Items for Systematic Reviews and Meta-Analyses Extension for Scoping Reviews
