# Supplementary material for: External context in individual placement and support implementation: a scoping review with abductive thematic analysis
Source: Implement Sci. 2023 Nov 9;18:61. doi: 10.1186/s13012-023-01316-w (PMC10636871; doi:10.1186/s13012-023-01316-w)
Supplement: Supplementary file 2 — Additional file 2. List of references for included studies. [file 13012_2023_1316_MOESM2_ESM.docx]

# **Additional file 2.** List of references for included studies.

i1. Bakkeli V, Breit E. From “what works” to “making it work”: A practice perspective on evidence‐based standardization in frontline service organizations. Social Policy and Administration. 2022;56(1):87–102.

i2. Becker DR, Torrey WC, Toscano R, Wyzik PF, Fox TS. Building recovery-oriented services: Lessons from implementing Individual Placement and Support (IPS) in community mental health centers. Psychiatric Rehabilitation Journal. 1998;22(1):51–4.

i3. Becker DR, Xie H, McHugo GJ, Halliday J, Martinez RA. What predicts supported employment program outcomes? Community Mental Health Journal. 2006;42(3):303–13.

i4. Becker DR, Baker SR, Carlson L, Flint L, Howell R, Lindsay S, et al. Critical strategies for implementing supported employment. Journal of Vocational Rehabilitation. 2007;27(1):13–20.

i5. Bejerholm U, Larsson L, Hofgren C. Individual placement and support illustrated in the Swedish welfare system: A case study. Journal of Vocational Rehabilitation. 2011;35(1):59–72.

i6. Bergmark M, Bejerholm U, Urban Markström. Critical Components in Implementing Evidence‐based Practice: A Multiple Case Study of Individual Placement and Support for People with Psychiatric Disabilities. Social Policy and Administration. 2018;52(3):790–808.

i7. Bergmark M, Bejerholm U, Markstrom U. Implementation of evidence-based interventions: Analyzing critical components for sustainability in community mental health services. Social Work in Mental Health. 2019;17(2):129–48.

i8. Bond G, McHugo G, Becker D, Rapp C, Whitley R. Fidelity of supported employment: Lessons learned from the National Evidence Based Practice Project. Psychiatric Rehabilitation Journal. 2008;31(4):300–5

i9. Bond GR, Johnson-Kwochka AV, Becker DR, Drake RE, Greene MA. Sustaining and expanding evidence-based supported employment: The role of state leaders participating in a learning community. Administration and Policy in Mental Health and Mental Health Services Research. 2017;44(3):320–30.

i10. Bond GR, Johnson-Kwochka AV, Pogue JA, Langfitt Reese S, Becker DR, Drake RE. A Tale of Four States: Factors Influencing the Statewide Adoption of IPS. Adm Policy Ment Health. 2021;48(3):528–38.

i11. Bonfils IS. Implementing the Individual Placement and Support approach in institutional settings for employment and mental health services – perceptions and challenges from a case study in Denmark. European Journal of Social Work. 2022 May;25(3):471–84.

i12. Boyce M, Secker J, Floyd M, Grove B, Johnson R, Schneider J, et al. Factors influencing the delivery of evidence-based supported employment in England. Psychiatric Rehabilitation Journal. 2008;31(4):360–6.

i13. Campbell K, Bond GR, Gervey R, Pascaris A, Tice S, Revell G. Does type of provider organization affect fidelity to evidence-based supported employment? Journal of Vocational Rehabilitation. 2007;27(1):3–11.

i14. Carlsson Stylianides K, Bejerholm U, Denvall V, Knutagård M, Johanson S. Implementation structures at work. Exploring implementation and de‐implementation attempts regarding Housing First and Individual Placement and Support. Social Policy & Administration. 2022;1.

i15. Cohen DA, Klodnick VV, Stevens L, Fagan MA, Spencer ES. Implementing adapted Individual Placement and Support (IPS) supported employment for transition-age youth in Texas. Community Mental Health Journal. 2020;56(3):513–23.

i16. Corbiere M, Lanctot N, Lecomte T, Latimer E, Goering P, Kirsh B, et al. A Pan-Canadian Evaluation of Supported Employment Programs Dedicated to People with Severe Mental Disorders. Community Mental Health Journal. 2010;46(1):44–55.

i17. de Greef V. Analysis of barriers and facilitators of existing programs in Belgium for the purpose of implementing individual placement and support (IPS). Psychiatric Rehabilitation Journal. 2020;43(1):18–23.

i18. Gowdy E, Carlson L, Rapp C. Organizational factors differentiating high performing from low performing supported employment programs. Psychiatric Rehabilitation Journal. 2004;28(2):150–6.

i19. Hamilton A, Cohen A, Glover D, Whelan F, Chemerinski E, McNagny K, et al. Implementation of Evidence-Based Employment Services in Specialty Mental Health. Health Services Research. 2013;48(6):2224–44.

i20. Hasson H, Andersson M, Bejerholm U. Barriers in implementation of evidence-based practice: Supported employment in Swedish context. J Health Organ Manag. 2011;25(3):332–45.

i21. Hilarion P, Koatz D, Bonet P, Cid J, Pinar I, Otin JM, et al. Implementation of the individual placement and support pilot program in Spain. Psychiatric Rehabilitation Journal. 2020;43(1):65–71.

i22. Hillborg H, Bergmark M, Bejerholm U. Implementation of individual placement and support in a first‐episode psychosis unit: A new way of working. Social Policy and Administration. 2021;55(1):51–64.

i23. Hutchinson J, Gilbert D, Papworth R, Boardman J. Implementing Supported Employment. Lessons from the Making IPS Work Project. Int J Environ Res Public Health. 2018;15(7).

i24. Isett K, Burnam M, Coleman-Beattie B, Hyde P, Morrissey J, Magnabosco J, et al. The state policy context of implementation issues for evidence-based practices in mental health. Psychiatric Services. 2007;58(7):914–21.

i25. Johnson-Kwochka A, Bond GR, Becker DR, Drake RE, Greene MA. Prevalence and quality of individual placement and support (IPS) supported employment in the United States. Administration and Policy in Mental Health and Mental Health Services Research. 2017;44(3):311–9.

i26. Knaeps J, DeSmet A, Van Audenhove C. The IPS fidelity scale as a guideline to implement Supported Employment. Journal of Vocational Rehabilitation. 2012;37(1):13–23.

i27. Latimer E, Bordeleau F, Methot C, Barrie T, Ferkranus A, Lurie S, et al. Implementation of supported employment in the context of a national Canadian program: Facilitators, barriers and strategies. Psychiatric Rehabilitation Journal. 2020;43(1):2–8.

i28. Lockett H, Waghorn G, Kydd R. Policy barriers to evidence-based practices in vocational rehabilitation for people with psychiatric disabilities in New Zealand. Work-A Journal Of Prevention Assessment & Rehabilitation. 2018;60(3):421–35.

i29. Marshall T, Rapp C, Becker D, Bond G. Key factors for implementing supported employment. Psychiatric Services. 2008;59(8):886–92.

i30. Menear M, Reinharz D, Corbiere M, Houle N, Lanctot N, Goering P, et al. Organizational analysis of Canadian supported employment programs for people with psychiatric disabilities. Social Science & Medicine. 2011;72(7):1028–35.

i31. Moe C, Brinchmann B, Rasmussen L, Brandseth OL, McDaid D, Killackey E, et al. Implementing individual placement and support (IPS): The experiences of employment specialists in the early implementation phase of IPS in Northern Norway. The IPSNOR study. BMC Psychiatry. 2021;21.

i32. Moe C, Brinchmann B, Borg M, McDaid D, Rinaldi M, Killackey E, et al. Implementing individual placement and support in Norway. From vocational rehabilitation to an employment scheme. Social Policy & Administration. 2022;1.

i33. Noel VA, Bond GR, Drake RE, Becker DR, McHugo GJ, Swanson SJ, et al. Barriers and facilitators to sustainment of an evidence-based supported employment program. Administration and Policy in Mental Health and Mental Health Services Research. 2017;44(3):331–8.

i34. Noel VA, Oulvey E, Drake RE, Bond GR, Carpenter-Song EA, DeAtley B. A preliminary evaluation of individual placement and support for youth with developmental and psychiatric disabilities. Journal of Vocational Rehabilitation. 2018;48(2):249–55.

i35. Oldman J, Thomson L, Calsaferri K, Luke A, Bond GR. A case report of the conversion of sheltered employment to evidence-based supported employment in Canada. Psychiatric Services. 2005;56(11):1436–40.

i36. Parletta VA, Waghorn G. The financial viability of evidence-based supported employment for people with mental illnesses in a blended funding system. Journal of Vocational Rehabilitation. 2016;44(2):227–41.

i37. Patel SR, Margolies PJ, Covell NH, Lipscomb C, Dixon LB. Using Instructional Design, Analyze, Design, Develop, Implement, and Evaluate, to Develop e-Learning Modules to Disseminate Supported Employment for Community Behavioral Health Treatment Programs in New York State. Front Public Health. 2018;6:113.

i38. Pogoda T, Cramer I, Rosenheck R, Resnick S. Qualitative Analysis of Barriers to Implementation of Supported Employment in the Department of Veterans Affairs. Psychiatric Services. 2011;62(11):1289–95.

i39. Pogue JA, Bond GR, Drake RE, Becker DR, Logsdon SM. Growth of IPS Supported Employment Programs in the United States: An Update. Psychiatr Serv. 2021;appips202100199.

i40. Priest B, Lockett H. Working at the interface between science and culture: The enablers and barriers to individual placement and support implementation in Aotearoa/New Zealand. Psychiatric Rehabilitation Journal. 2020;43(1):40–52.

i41. Rapp C, Etzel-Wise D, Marty D, Coffman M, Carlson L, Asher D, et al. Barriers to Evidence-Based Practice Implementation: Results of a Qualitative Study. Community Mental Health Journal. 2010;46(2):112–8.

i42. Roeg D, de Winter L, Bergmans C, Couwenbergh C, McPherson P, Killaspy H, et al. IPS in Supported Housing: Fidelity and Employment Outcomes Over a 4 Year Period. Front Psychiatry. 2020;11:622061.

i43. Salkever D, Abrams M, Baier K, Gibbons B. Impacting entry into evidence-based supported employment: A population-based empirical analysis of a statewide public mental health program in Maryland. Administration and Policy in Mental Health and Mental Health Services Research. 2018;45(2):328–41.

i44. Schneider J, Akhtar A. Implementation of individual placement and support: The Nottingham experience. Psychiatric Rehabilitation Journal. 2012;35(4):325–32.

i45. Sharek D, Lally N, Brennan C, Higgins A. “These are people just like us who can work”: Overcoming clinical resistance and shifting views in the implementation of Individual Placement and Support (IPS). Adm Policy Ment Health. 2022;49(5):848–60.

i46. Stirling Y, Higgins K, Petrakis M. Challenges in implementing individual placement and support in the Australian mental health service and policy context. Aust Health Rev. 2018;42(1):82–8.

i47. Swain K, Whitley R, McHugo G, Drake R. The Sustainability of Evidence-Based Practices in Routine Mental Health Agencies. Community Mental Health Journal. 2010;46(2):119–29.

i48. Swanson SJ, Courtney CT, Meyer RH, Reeder SA. Strategies for integrated employment and mental health services. Psychiatric Rehabilitation Journal. 2014;37(2):86–9.

i49. Talbot E, Bird Y, Russell J, Sahota K, Schneider J, Khalifa N. Implementation of individual placement and support (IPS) into community forensic mental health settings: Lessons learned. The British Journal of Occupational Therapy. 2018;81(6):338–47.

i50. Thomas JR, Fraser VV. Implementing evidence-based supported employment in a recovery-oriented mental health agency. American Journal of Psychiatric Rehabilitation. 2009;12(2):143–60.

i51. van Duin D, Franx G, Van Wijngaarden B, Van Der Gaag M, Van Weeghel J, Slooff C, et al. Bridging the science-to-service gap in schizophrenia care in the Netherlands: The Schizophrenia Quality Improvement Collaborative. International Journal for Quality in Health Care. 2013;25(6):626–32.

i52. van Duin D, van Wamel A, de Winter L, Kroon H, Veling W, van Weeghel J. Implementing evidence-based interventions to improve vocational recovery in early psychosis: A quality-improvement report. Psychiatric Services. 2021;72(10):1168–77.

i53. van Erp NHJ, Giesen FBM, van Weeghel J, Kroon H, Michon HWC, Becker D, et al. A multisite study of implementing supported employment in the Netherlands. Psychiatric Services. 2007;58(11):1421–6.

i54. van Weeghel J, Bergmans C, Couwenbergh C, Michon H, de Winter L. Individual placement and support in the Netherlands: Past, present, and future directions. Psychiatric Rehabilitation Journal. 2020;43(1):24–31.

i55. Vukadin M, Schaafsma FG, Westerman MJ, Michon HWC, Anema JR. Experiences with the implementation of Individual Placement and Support for people with severe mental illness: A qualitative study among stakeholders. BMC Psychiatry. 2018;18

i56. Vukadin M, Schaafsma FG, Michon HWC, de Maaker-Berkhof M, Anema JR. Experiences with individual placement and support and employment-A qualitative study among clients and employment specialists. BMC Psychiatry. 2021;21

i57. Waghorn G, Collister L, Killackey E, Sherring J. Challenges to implementing evidence-based supported employment in Australia. Journal of Vocational Rehabilitation. 2007;27(1):29–37.

i58. Waghorn G, Killackey E, Dickson P, Brock L, Skate C. Evidence-based supported employment for people with psychiatric disabilities in Australia: Progress in the past 15 years. Psychiatric Rehabilitation Journal. 2020;43(1):32–9.

i59. Zhen‐Duan J, Chary A, NeMoyer A, Fukuda M, Markle SL, Hoyos M, et al. Key stakeholder perspectives on the use of research about supported employment for racially and ethnically diverse patients with mental illness in the United States. Health Services Research. 2022;57(S1):95–104.
