# Supplementary material for: External context in individual placement and support implementation: a scoping review with abductive thematic analysis
Source: Implement Sci. 2023 Nov 9;18:61. doi: 10.1186/s13012-023-01316-w (PMC10636871; doi:10.1186/s13012-023-01316-w)
Supplement: Supplementary file 3 — Additional file 3. List of references for excluded full-text studies. [file 13012_2023_1316_MOESM3_ESM.docx]

# **Additional file 3.** List of references for excluded full-text studies.

e1. Al-Abdulmunem M, Drake RE, Carpenter-Song E. Evidence-based supported employment in the rural United States: Challenges and adaptations. Psychiatric Services. 2021;72(6):712–5.

e2. Becker DR, Courtney C, Reese SL. Update on funding IPS supported employment services in the IPS learning community. Psychiatric Rehabilitation Journal. 2022;(Ali, M., Schur, L., Blanck, P. (2011). What types of jobs do people with disabilities want?. Journal of Occupational Rehabilitation, 21, 2, 199-210. http://dx.doi.org/10.1007/s10926-010-9266-0Becker, D. R., Drake, R. E., Bond, G. R. (2014). The IPS su):No-Specified.

e3. Becker DR, Drake RE, Bond GR. The IPS supported employment learning collaborative. Psychiatric Rehabilitation Journal. 2014;37(2):79–85.

e4. Becker DR, Lynde D, Swanson SJ. Strategies for state-wide implementation of supported employment: The Johnson & Johnson-Dartmouth Community Mental Health Program. Psychiatric Rehabilitation Journal. 2008;31(4):296–9.

e5. Boardman J, Rinaldi M. Difficulties in implementing supported employment for people with severe mental health problems. The British Journal of Psychiatry. 2013;203(4):247–9.

e6. Bond GR, Drake RE, Becker DR. Beyond evidence-based practice: Nine ideal features of a mental health intervention. Research on Social Work Practice. 2010;20(5):493–501.

e7. Bond GR, Drake RE, Becker DR, Noel VA. The IPS learning community: A longitudinal study of sustainment, quality, and outcome. Psychiatric Services. 2016;67(8):864–9.

e8. Bond GR, Drake RE, Becker DR, Noel V. Sustaining Individual Placement and Support (IPS) services: the IPS Learning Community. World Psychiatry. 2016;15(1):81–3.

e9. Booth D, Francis S, Mcivor N, Hinson P, Barton B. Severe mental illness & employment: cost-benefit analysis and dynamics of decision making. Mental Health and Social Inclusion. 2014;18(4):215–23.

e10. Boycott N, Schneider J, Osborne M. Creating a culture of employability in mental health. Mental Health and Social Inclusion. 2014;18(1):29–34.

e11. Brinchmann B, Widding-Havneraas T, Modini M, Rinaldi M, Moe CF, McDaid D, et al. A meta-regression of the impact of policy on the efficacy of individual placement and support. Acta Psychiatrica Scandinavica. 2020;141(3):206–20.

e12. Chalamat M, Mihalopoulos C, Carter R, Vos T. Assessing cost-effectiveness in mental health: Vocational rehabilitation for schizophrenia and related conditions. Australian and New Zealand Journal of Psychiatry. 2005;39(8):693–700.

e13. Christensen TN, Kruse M, Hellstrom L, Eplov LF. Cost-utility and cost-effectiveness of individual placement support and cognitive remediation in people with severe mental illness: Results from a randomized clinical trial. European Psychiatry. 2020;64(Ankjaer-Jensen, A., Rosling, P., Bilde, L. (2006). Variable prospective financing in the Danish hospital sector and the development of a Danish case-mix system. Health Care Manag Sci. 2006;9(3):259-68. doi: http://link.springer.com/10.1007/s10729-006-90).

e14. Clark RE, Xie H, Becker DR, Drake RE. Benefits and costs of supported employment from three perspectives. The Journal of Behavioral Health Services & Research. 1998;25(1):22–35.

e15. Cocks E, Boaden R. Evaluation of an employment program for people with mental illness using the Supported Employment Fidelity Scale. Australian Occupational Therapy Journal. 2009;56(5):300–6.

e16. Costa M, Baker M, Davidson L, Giard J, Guillorn L, Ibanez AG, et al. Provider perspectives on employment for people with serious mental illness. International Journal of Social Psychiatry. 2017;63(7):632–40.

e17. Dawson S, Muller J, Renigers V, Varona L, Kernot J. Consumer, health professional and employment specialist experiences of an individual placement and support programme. Scand J Occup Ther. 2021;28(6):433–45.

e18. Dixon L, Hoch JS, Clark R, Bebout R, Drake R, McHugo G, et al. Cost-effectiveness of two vocational rehabilitation programs for persons with severe mental illness. Psychiatric Services. 2002;53(9):1118–24.

e19. Drake RE, Becker DR, Bond GR. Growth and sustainment of individual placement and support. Psychiatric Services. 2020;71(10):1075–7.

e20. Ellison ML, Klodnick VV, Bond GR, Krzos IM, Kaiser SM, Fagan MA, et al. Adapting supported employment for emerging adults with serious mental health conditions. The Journal of Behavioral Health Services & Research. 2015;42(2):206–22.

e21. Evans LJ, Bond GR. Expert ratings on the critical ingredients of supported employment for people with severe mental illness. Psychiatric Rehabilitation Journal. 2008;31(4):318–31.

i22. Ferguson KM. Using the Social Enterprise Intervention (SEI) and Individual Placement and Support (IPS) models to improve employment and clinical outcomes of homeless youth with mental illness. Social Work in Mental Health. 2013;11(5):473–95.

e23. Fioritti A, Burns T, Hilarion P, van Weeghel J, Cappa C, Sunol R, et al. Individual placement and support in Europe. Psychiatric Rehabilitation Journal. 2014;37(2):123–8.

e24. Fleming C, Curtis R, Martin ED, Kraska M, Shippen M, Varda K. Perceptions and practices of mental health professionals regarding the employment of people with serious mental illness. Journal of Vocational Rehabilitation. 2019;50(1):39–48.

e25. Fraser VV, Jones AM, Frounfelker R, Harding B, Hardin T, Bond GR. VR closure rates for two vocational models. Psychiatric Rehabilitation Journal. 2008;31(4):332–9.

e26. Fyhn T, Ludvigsen K, Reme SE, Schaafsma F. A structured mixed method process evaluation of a randomized controlled trial of Individual Placement and Support (IPS). Implement Sci Commun. 2020;1:95.

e27. Giesen F, van Erp N, van Weeghel J, Michon H, Kroon H. [The implementation of Individual Placement and Support in the Netherlands]. Tijdschr Psychiatr. 2007;49(9):611–21.

e28. Hayashi T, Yamaguchi S, Sato S. Implementing the individual placement and support model of supported employment in Japan: Barriers and strategies. Psychiatric Rehabilitation Journal. 2020;43(1):53–9.

e29. Hegelstad W ten V, Joa I, Heitmann L, Johannessen JO, Langeveld J. Job- and schoolprescription: A local adaptation to individual placement and support for first episode psychosis. Early Intervention in Psychiatry. 2019;13(4):859–66.

e30. Hellström L, Kruse M, Christensen TN, Trap Wolf R, Eplov LF. Cost-effectiveness analysis of a supported employment intervention for people with mood and anxiety disorders in Denmark - the IPS-MA intervention. Nord J Psychiatry. 2021;75(5):389–96.

e31. Heslin M, Howard L, Leese M, McCrone P, Rice C, Jarrett M, et al. Randomized controlled trial of supported employment in England: 2 year follow-up of the Supported Work and Needs (SWAN) study. World Psychiatry. 2011;10(2):132–7.

e32. Hoffmann H, Jackel D, Glauser S, Mueser KT, Kupper Z. Long-term effectiveness of supported employment: 5-year follow-up of a randomized controlled trial. The American Journal of Psychiatry. 2014;171(11):1183–90.

e33. Howard LM, Heslin M, Leese M, McCrone P, Rise C, Jarrett M, et al. Supported Employment: Randomized Controlled Trial. The British Journal of Psychiatry. 2010;196(5):404–11.

e34. Kern RS, Zarate R, Glynn SM, Turner LR, Smith KM, Mitchell SS, et al. A demonstration project involving peers as providers of evidence-based, supported employment services. Psychiatric Rehabilitation Journal. 2013;36(2):99–107.

e35. Khalifa N, Hadfield S, Thomson L, Talbot E, Bird Y, Schneider J, et al. Barriers and facilitators to the implementation of individual placement and support (IPS) for patients with offending histories in the community: The United Kingdom experience. The British Journal of Occupational Therapy. 2020;83(3):179–90.

e36. Killackey E, Waghorn G. The challenge of integrating employment services with public mental health services in Australia: Progress at the first demonstration site. Psychiatric Rehabilitation Journal. 2008;32(1):63–6.

e37. Knapp M, Patel A, Curran C, Latimer E, Catty J, Becker T, et al. Supported employment: cost-effectiveness across six European sites. World Psychiatry. 2013;12(1):60–8.

e38. Larson JE, Sheehan L, Ryan C, Lemp S, Drandorff L. Practitioner perspectives on Individual Placement and Support (IPS) for individuals with serious mental illness. Journal of Vocational Rehabilitation. 2014;41(3):225–35.

e39. Latimer EA, Bush PW, Becker DR, Drake RE, Bond GR. The Cost of High-Fidelity Supported Employment Programs for People With Severe Mental Illness. Psychiatric Services. 2004;55(4):401–6.

e40. Lloyd C, King R. Implementation of supported employment: What are the implications for clinical services? Journal of Rehabilitation. 2012;78(1):25–9.

e41. Lockett H. Looking for the leadership to successfully implement evidence-based supported employment in mental health services - what has leadership theory got to offer? International Journal of Leadership in Public Services. 2009;5(2):14–8.

e42. Lord SE, McGurk SR, Nicholson J, Carpenter-Song EA, Tauscher JS, Becker DR, et al. The potential of technology for enhancing individual placement and support supported employment. Psychiatric Rehabilitation Journal. 2014;37(2):99–106.

e43. Luciano A, Bond GR, Drake RE, Becker DR. Is high fidelity to supported employment equally attainable in small and large communities? Community Mental Health Journal. 2014;50(1):46–50.

e44. Markström U, Svensson B, Bergmark M, Hansson L, Bejerholm U. What influences a sustainable implementation of evidence-based interventions in community mental health services? Development and pilot testing of a tool for mapping core components. J Ment Health. 2018;27(5):395–401.

e45. Meisler N, Williams O. Replicating effective supported employment models for adults with psychiatric disabilities. Psychiatric Services. 1998;49(11):1419–21.

e46. Melleney L, Kendall T. Individual Placement and Support (IPS) in England. Psychiatric Rehabilitation Journal. 2020;43(1):76–8.

e47. Morris A, Waghorn G, Robson E, Moore L, Edwards E. Implementation of evidence-based supported employment in regional Australia. Psychiatric Rehabilitation Journal. 2014;37(2):144–7.

e48. Murphy A, Mullen M, Spagnolo A. Enhancing Individual Placement and Support: Promoting Job Tenure by Integrating Natural Supports and Supported Education. American Journal of Psychiatric Rehabilitation. 2005;8(1):37–61.

e49. Nuechterlein KH, Subotnik KL, Turner LR, Ventura J, Becker DR, Drake RE. Individual placement and support for individuals with recent-onset schizophrenia: Integrating supported education and supported employment. Psychiatric Rehabilitation Journal. 2008;31(4):340–9.

e50. Oulvey E, Carpenter-Song EA, Swanson SJ. Principles for enhancing the role of state vocational rehabilitation in IPS-supported employment. Psychiatric Rehabilitation Journal. 2013;36(1):4–6.

e51. Perkins R, Patel R, Willett A, Chisholm L, Rinaldi M. Individual placement and support: cross-sectional study of equality of access and outcome for Black, Asian and minority ethnic communities. BJPsych Bull. 2022;46(1):10–5.

e52. Porteous N, Waghorn G. Developing evidence-based supported employment services for young adults receiving public mental health services. New Zealand Journal of Occupational Therapy. 2009;56(1):34–9.

e53. Rinaldi M, Miller L, Perkins R. Implementing the individual placement and support (IPS) approach for people with mental health conditions in England. International Review of Psychiatry. 2010;22(2):163–72.

e54. Secker J. Supporting mental health service users back to work. Journal of Public Mental Health. 2009;8(3):38–45.

e55. Shepherd G, Lockett H, Bacon J, Grove B. Establishing IPS in clinical teams - Some key themes from a national implementation programme. Journal of Rehabilitation. 2012;78(1):30–6.

e56. Solar A. A supported employment linkage intervention for people with schizophrenia who want a chance to work. Australasian Psychiatry. 2014;22(3):245–7.

e57. Sveinsdottir V, Bull HC, Evensen S, Reme SE, Knutzen T, Lystad JU. A short history of individual placement and support in Norway. Psychiatric Rehabilitation Journal. 2020;43(1):9–17.

e58. Waghorn G, Lockett H, Bacon J, Gorman P, Durie S. The importance of leadership in implementing evidence-based supported employment services for people with severe mental illness. International Journal of Leadership in Public Services. 5:51–6.

e59. Whitworth A. The economic case for well-considered investment in health-related employment support: Costs and savings of alternative modified Individual and Placement Support (IPS) models. Disability and Health Journal. 2018;11(4):568–75.

e60. Whitworth A. Mainstreaming Effective Employment Support for Individuals with Health Conditions: An Analytical Framework for the Effective Design of Modified Individual Placement and Support (IPS) Models. Social Policy and Society. 2019;18(4):517–33.
